# Giant thyroglossal duct cyst causing hoarseness due to laryngeal compression in an elderly patient: a case report

**DOI:** 10.3389/fsurg.2026.1770599

**Published:** 2026-06-15

**Authors:** Caini Peng, Bingyan Hao, Aoshuang Chang

**Affiliations:** 1 Clinical Medical College of Guizhou Medical University, Guiyang, Guizhou, China; 2 Department of Otorhinolaryngology Head and Neck Surgery, Affiliated Hospital of Guizhou Medical University, Guiyang, Guizhou, China

**Keywords:** case report, elderly, hoarseness, sistrunk procedure, thyroglossal duct cyst

## Abstract

Thyroglossal duct cysts (TGDCs) are among the most common congenital neck anomalies and account for a substantial proportion of congenital neck lesions. Although they usually present in childhood, often within the first decade of life, first presentation in elderly patients is distinctly uncommon. This report presents a case of a giant TGDC causing hoarseness due to laryngeal compression in an elderly patient treated at the Department of Otorhinolaryngology Head and Neck Surgery of the Affiliated Hospital of Guizhou Medical University. The patient presented with a 2-year history of an anterior midline neck mass and a 1-year history of progressive enlargement with hoarseness, occasional phonation difficulty, foreign-body sensation, and swallowing obstruction. Preoperative ultrasound and neck CT showed a giant cystic-solid midline cervical lesion surrounding the hyoid bone and extending inferiorly with compression and narrowing of the adjacent laryngeal cavity. CT also identified an incidental small nodule in the lower region of the left thyroid lobe. Preoperative FNAC and detailed thyroid function test results were not documented in the available record and are acknowledged as limitations. The patient underwent open excision based on the Sistrunk principle with partial hyoid bone resection. Histopathological examination supported TGDC with old hemorrhage and cholesterol crystal formation. Postoperatively, the patient's hoarseness and swallowing-related symptoms resolved, and no abnormality was observed on 3-month follow-up laryngoscopy.

## Introduction

Thyroglossal duct cysts (TGDCs) arise from incomplete obliteration of the thyroglossal duct during early embryonic thyroid development (1).They are the most common congenital cervical anomaly, accounting for approximately 70% of congenital neck lesions, and are present in about 7% of the general population. Most clinically recognized cases present in childhood or adolescence; however, up to one-third are reported in patients aged 20 years or older. In contrast, first presentation in elderly patients is distinctly uncommon, with older reports estimating that only approximately 0.6% of cases occur in patients older than 60 years (2). Therefore, the rarity of TGDC at first presentation in elderly patients represents a major epidemiological strength of the present case. These cysts may occur anywhere along the thyroglossal duct tract, from the foramen cecum to the lower anterior neck. Anatomically, approximately 20%–25% occur at the suprahyoid level, 15%–20% at the level of the hyoid, and 25%–65% at the infrahyoid level, making the infrahyoid region the most common site of involvement. Clinically, they typically present as painless, mobile midline neck masses (3). The primary treatment for TGDCs is surgical excision, with the Sistrunk procedure (resection of the cyst along with the mid-portion of the hyoid bone and the connecting tract to the foramen cecum) being the preferred approach due to its low recurrence rate (4, 5). Here, we report a giant infrahyoid TGDC in a 65-year-old man presenting with hoarseness due to laryngeal compression, an unusual clinical scenario that further expands the spectrum of TGDC presentation in older adults. This case also highlights the importance of careful diagnostic assessment in elderly patients with cystic neck masses, including differentiation from laryngeal lesions, cystic metastatic lymphadenopathy, ectopic thyroid tissue, and rare TGDC-associated carcinoma.

## Case report

**Figure 1:**
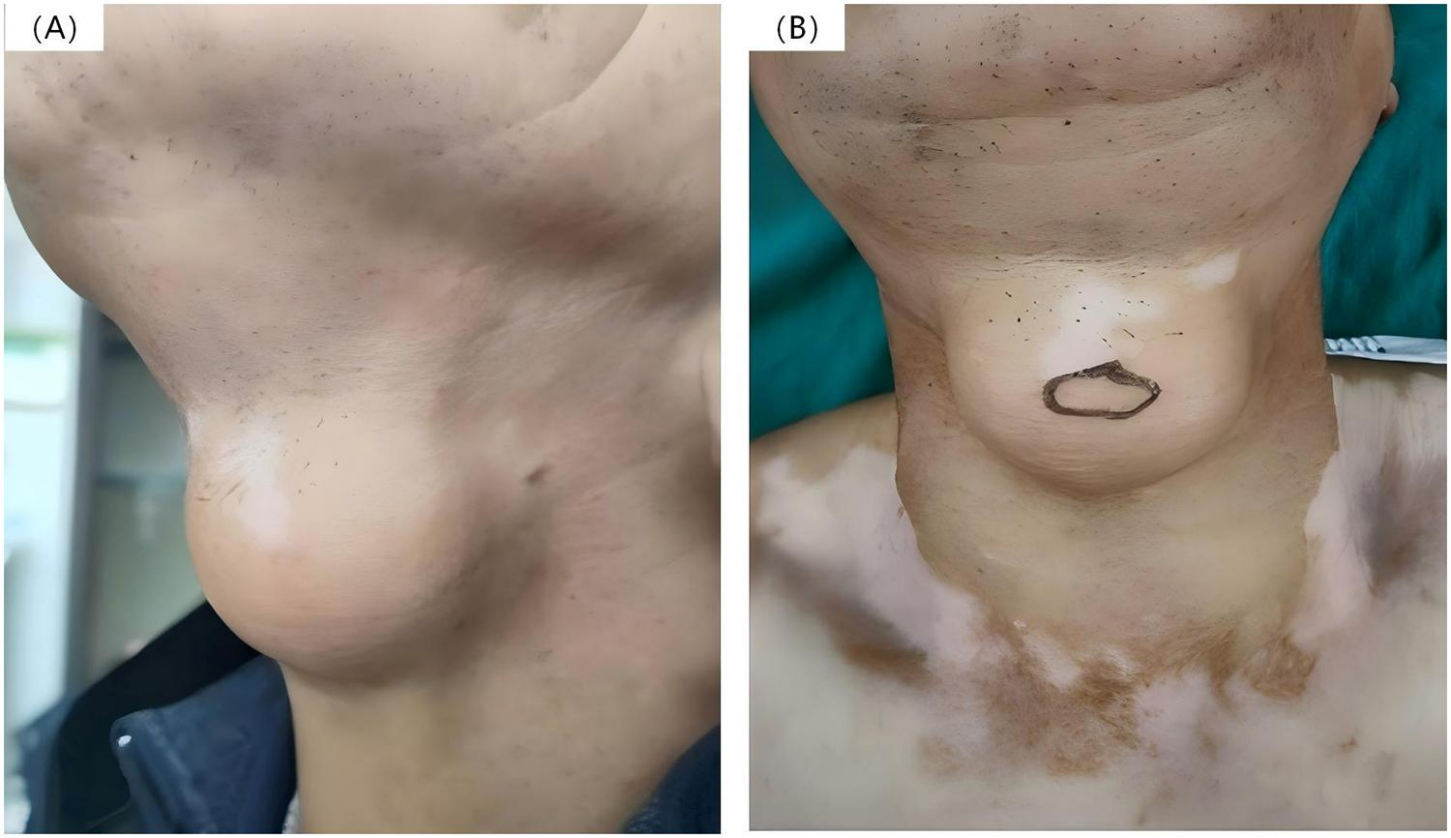
Preoperative clinical appearance of the anterior neck mass. **(A)** Lateral view showing a large, smooth, rounded swelling in the anterior midline neck, predominantly located at the level of and below the hyoid bone. **(B)** Frontal view showing a well-circumscribed midline anterior neck mass, with preoperative skin marking over the most prominent area.

A 65-year-old man presented with a 2-year history of an anterior midline neck mass and a 1-year history of progressive enlargement accompanied by hoarseness. Two years before admission, he incidentally noticed a mass in the anterior midline neck, without pain, fever, dyspnea, or dysphagia. One year before admission, the mass further enlarged and was accompanied by hoarseness, occasional phonation difficulty, a foreign-body sensation in the throat, and a sensation of swallowing obstruction. He denied dyspnea, fever, and significant weight loss. He also denied previous episodes suggestive of cyst infection, such as painful swelling, local erythema, fever, or spontaneous discharge. Before admission to our institution, he had been evaluated at a local hospital and was advised to seek surgical treatment at a higher-level center; no prior intervention for the neck mass had been performed. His past medical history was significant for vitiligo for more than 3 years without regular treatment. He denied hypertension, diabetes mellitus, heart disease, kidney disease, tuberculosis, viral hepatitis, previous surgery, trauma, blood transfusion, long-term medication use, and drug or food allergies. He had a drinking history of more than 30 years, approximately 100 g/day, and a smoking history of more than 30 years, although the cumulative amount could not be accurately estimated. His family history was unremarkable. Physical examination revealed an approximately 5 cm × 5 cm smooth and firm mass in the anterior midline neck at the level of the hyoid bone. The mass was well demarcated, non-tender, and moved with swallowing. The overlying skin was intact (Figure 1). The detailed clinical timeline of diagnosis and treatment is summarized in Table 1.

**Table 1:** Timeline of the episode of care.

Time point	Clinical event	Diagnostic/Therapeutic action	Outcome
2 years before admission	Incidentally noticed a midline anterior neck mass	No medical intervention	No pain, fever, dyspnea, or dysphagia
1 year before admission	Progressive enlargement of the mass with hoarseness, occasional phonation difficulty, foreign-body sensation, and swallowing obstruction	Evaluation at a local hospital	Referred to a higher-level center for surgical treatment
At admission	Persistent anterior midline neck mass with hoarseness and swallowing-related symptoms	Physical examination, laryngoscopic evaluation, cervical ultrasound, and neck CT	TGDC considered the leading diagnosis; mixed laryngocele, primary laryngeal lesion, and malignancy considered in the differential diagnosis
21-Mar-24	Surgical treatment	Open excision based on the Sistrunk principle with partial hyoid bone resection	Complete excision of the cyst; approximately 30 mL of coffee-colored fluid aspirated intraoperatively; estimated blood loss approximately 10 mL
First 14 h postoperatively	Early postoperative monitoring	Negative-pressure drainage	22 mL bloody drainage
Postoperative day 2	Continued postoperative monitoring	Negative-pressure drainage	42 mL drainage
Postoperative day 3	Clinical improvement	Drain removal and discharge	4 mL drainage; discharged in stable condition
2 weeks postoperatively	Telephone follow-up	Symptom assessment	No hoarseness, phonation difficulty, foreign-body sensation, or swallowing obstruction
3 months postoperatively	Outpatient follow-up	Clinical examination and laryngoscopy	No symptoms; laryngoscopy showed no abnormalities

### Auxiliary examinations

**Figure 2:**
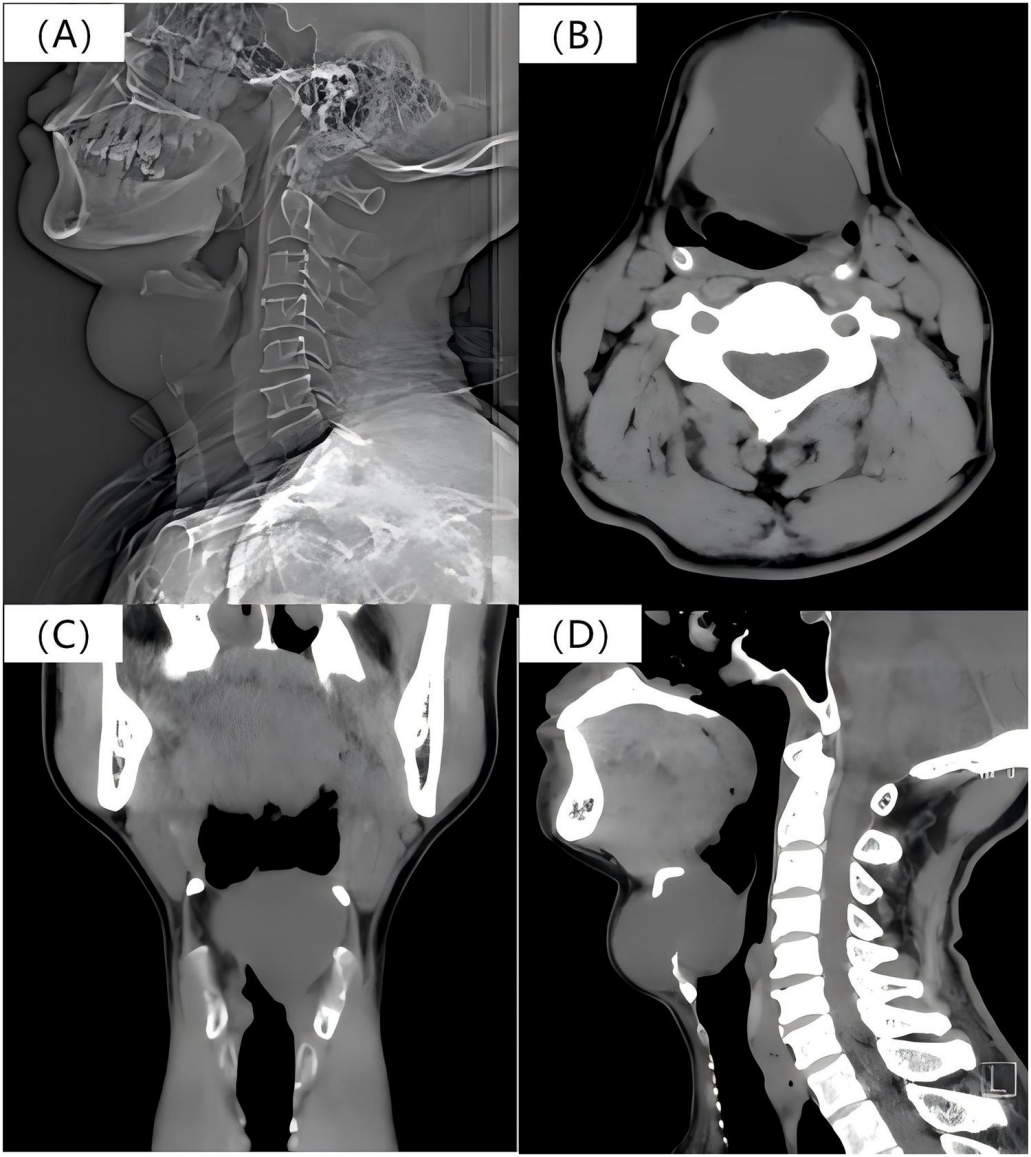
Preoperative computed tomography findings of the giant infrahyoid thyroglossal duct cyst. **(A)** Lateral scout view showing a large soft-tissue mass in the anterior neck. **(B)** Axial CT image showing a cystic low-density lesion inferior to the hyoid bone, with compression of the adjacent laryngeal cavity. **(C)** Coronal CT image showing the midline location of the lesion in the infrahyoid neck. **(D)** Sagittal CT image showing a large infrahyoid cystic lesion extending deeply and causing secondary compression and narrowing of the adjacent laryngeal cavity.

- Neck ultrasound: Cervical ultrasound showed multiple lymph nodes in bilateral neck levels I–IV, mainly in level II, without obvious abnormalities in morphology, internal structure, or blood-flow pattern. A cystic-solid lesion was detected in the anterior midline neck, measuring approximately 123 mm × 52 mm × 56 mm. The lesion was regular in shape, well circumscribed, and surrounded the hyoid bone, with a small amount of internal blood-flow signal on color Doppler imaging. These findings suggested the possibility of a TGDC.
- Neck CT: Neck CT demonstrated a cystic low-density lesion inferior to the hyoid bone, with an attenuation value of approximately 16 HU and a size of approximately 5.4 cm × 3.8 cm × 4.2 cm. The lesion was relatively regular in shape and well demarcated. It extended inferiorly to the level of the vocal cords and caused compression and narrowing of the adjacent laryngeal cavity. No obvious space-occupying lesion was identified in the nasopharyngeal or oropharyngeal region within the scanned field. CT also showed a small nodular shadow measuring approximately 14 mm × 12 mm in the lower region of the left thyroid lobe, with small calcifications. The imaging findings suggested a TGDC and also indicated the need for further thyroid nodule evaluation (Figure 2).
- Laryngoscopic evaluation: Indirect laryngoscopy showed a smooth epiglottis without congestion, symmetrical arytenoids without edema, normal abduction and adduction of both vocal cords, and no visible laryngeal neoplasm. A preoperative dynamic laryngoscopic examination reported a laryngeal mass or bulging lesion. In combination with CT findings, this laryngoscopic abnormality was interpreted as secondary supraglottic bulging caused by external compression rather than a primary laryngeal tumor (Figure 4).

Based on physical examination, laryngoscopic evaluation, cervical ultrasound, neck CT, intraoperative findings, and postoperative histopathological examination, TGDC was considered the leading diagnosis. The diagnostic work-up included physical examination, indirect and dynamic laryngoscopic evaluation, cervical ultrasound, neck CT, intraoperative exploration, and postoperative histopathological examination. The main diagnostic challenge in this case was that the patient presented with hoarseness and laryngeal compression, which could mimic a primary laryngeal lesion or mixed laryngocele. In addition, because the patient was elderly and had long-term smoking and alcohol exposure, laryngeal malignancy and cystic metastatic cervical lymphadenopathy also had to be considered. Diagnostic reasoning favored TGDC because the lesion was located in the anterior midline neck around the hyoid level, moved with swallowing, surrounded the hyoid bone on ultrasound, and appeared as a well-demarcated cystic low-density lesion on CT. CT demonstrated external compression and narrowing of the adjacent laryngeal cavity rather than a primary intralaryngeal lesion. Indirect laryngoscopy showed no visible laryngeal neoplasm and preserved bilateral vocal cord mobility. The differential diagnosis included mixed laryngocele, primary laryngeal tumor, cystic metastatic cervical lymph node, branchial cleft cyst, lymphatic malformation, dermoid or epidermoid cyst, ectopic thyroid tissue, and TGDC-associated carcinoma. Preoperative CT showed thyroid-related structures in the normal cervical anatomical region, but also identified a small nodular shadow measuring approximately 14 mm × 12 mm in the lower region of the left thyroid lobe, with small calcifications. Detailed thyroid function test results and dedicated thyroid nodule evaluation were not available in the scanned record; therefore, this point is acknowledged as a limitation. Preoperative FNAC was not documented in the available medical record. Given the patient's age, cystic neck mass, and incidental thyroid-region nodule, FNAC would have been useful for further preoperative risk stratification. In this case, surgery was performed because of the giant size of the lesion, progressive enlargement, hoarseness, swallowing discomfort, and significant laryngeal compression. Final histopathological examination supported TGDC with old hemorrhage and cholesterol crystal formation, without a reported histopathological diagnosis of carcinoma. The short-term prognosis was favorable because the patient's hoarseness, phonation difficulty, foreign-body sensation, and swallowing obstruction resolved after surgery, and follow-up laryngoscopy at 3 months showed no abnormalities. Longer follow-up remains necessary to monitor recurrence and the incidental thyroid-region nodule.

**Figure 3:**
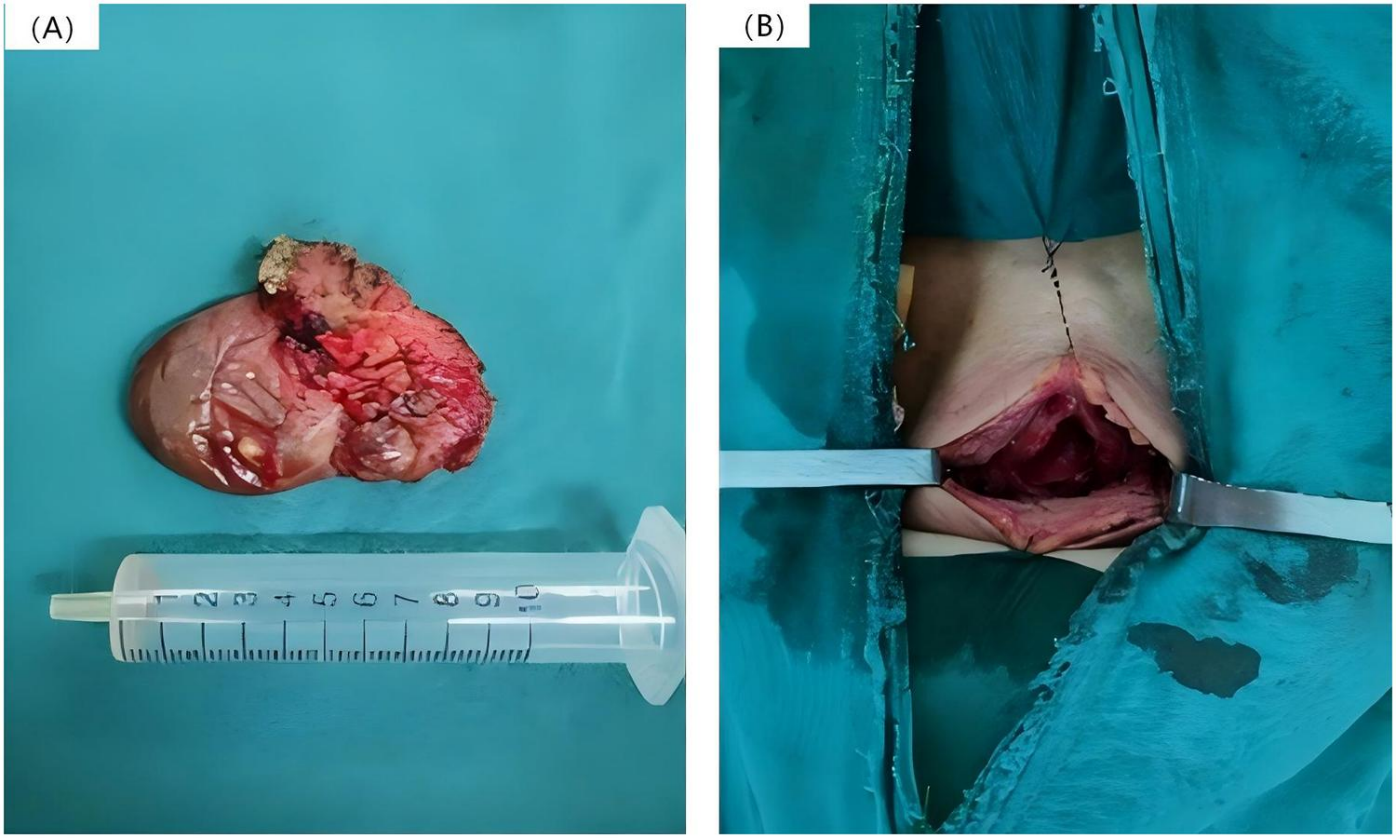
Gross specimen and intraoperative findings. **(A)** Gross specimen after excision, showing a large cystic lesion with a thickened wall and internal contents; a syringe is shown for size reference. **(B)** Intraoperative view after exposure of the lesion through an anterior cervical incision.

On March 21, 2024, the patient underwent open excision based on the Sistrunk principle under general anesthesia, including partial hyoid bone resection (Figure 3). A 5 cm transverse incision was made over the prominent mass below the hyoid bone. The skin and subcutaneous tissue were dissected, and the strap muscles were separated along the midline. A large intact cyst was identified deep to the strap muscles. To facilitate dissection, approximately 30 mL of coffee-colored fluid was aspirated using a 30 mL syringe, and the puncture site was ligated to prevent leakage. The cyst was closely attached to the hyoid bone, and a portion of the hyoid bone was resected. The lesion was dumbbell-shaped, partially extended posterior to the thyroid cartilage, and compressed the thyrohyoid membrane. Complete excision of the cyst was achieved. Hemostasis was secured, the surgical cavity was closed in layers, and a negative-pressure drain was inserted. The estimated intraoperative blood loss was approximately 10 mL.

**Figure 4:**
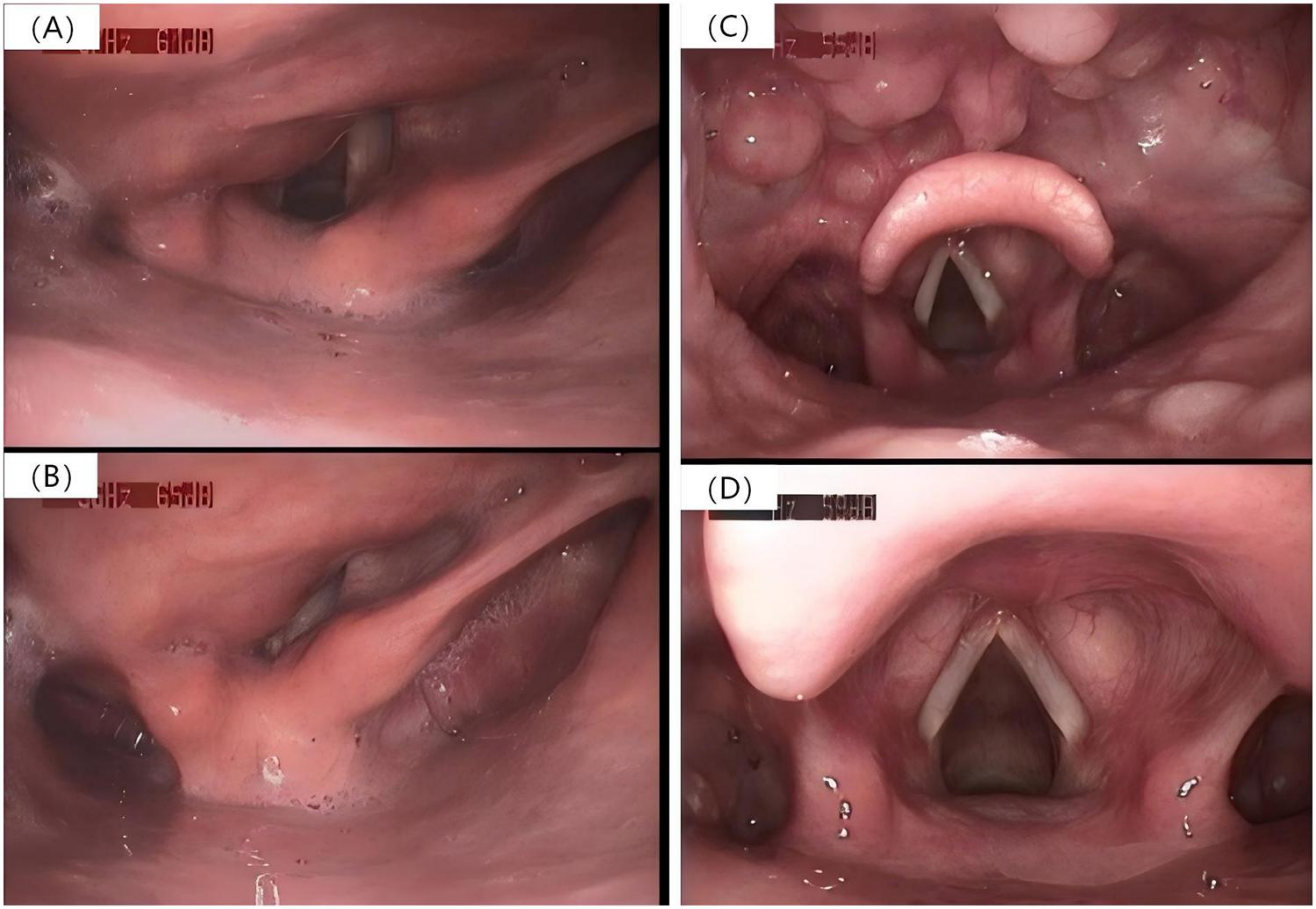
Preoperative and postoperative laryngoscopic findings. **(A,B)** Preoperative laryngoscopic views showing supraglottic bulging near the laryngeal surface of the epiglottis and ventricular fold region, consistent with secondary external compression in combination with CT findings. **(C,D)** Postoperative laryngoscopic views showing marked resolution of the previous supraglottic bulging and restoration of a normal laryngeal appearance without obvious residual abnormality.

### Histopathological findings

Gross pathological examination showed a gray-white cystic specimen measuring 5.5 cm × 4.5 cm × 2 cm, with a wall thickness of 0.1–0.3 cm. The cyst contained dark green, bean-dreg-like material. Histopathological examination was consistent with TGDC, accompanied by old hemorrhage and cholesterol crystal formation. No histopathological diagnosis of carcinoma was reported.

Postoperatively, the patient experienced marked relief of hoarseness and swallowing obstruction. The drainage volume was 22 mL of bloody fluid within the first 14 h, 42 mL on postoperative day 2, and 4 mL on postoperative day 3. The drain was removed on postoperative day 3, and the patient was discharged in stable condition. Telephone follow-up at 2 weeks revealed complete resolution of hoarseness, phonation difficulty, foreign-body sensation, and swallowing obstruction. At the 3-month follow-up visit, the patient remained asymptomatic, and laryngoscopy showed no abnormalities. No postoperative adverse or unanticipated events were documented during hospitalization or follow-up.

### Patient perspective

During follow-up, the patient reported complete resolution of hoarseness, phonation difficulty, foreign-body sensation, and swallowing obstruction after surgery. He expressed satisfaction with the postoperative improvement in voice and swallowing comfort.

## Discussion

This case is noteworthy because of the patient's advanced age, the giant size of the lesion, and its clinically significant extension resulting in laryngeal compression. In elderly patients, a cystic neck mass with hoarseness should not be assumed to be benign solely on the basis of its cystic appearance, particularly when there is a history of long-term smoking and alcohol exposure. TGDC is classically a lesion of childhood; however, adult presentation is not exceptional, with up to one-third of cases reported in patients aged 20 years or older. In contrast, first presentation in elderly patients remains distinctly uncommon, and older reports have estimated that only approximately 0.6% of TGDCs occur in patients older than 60 years. Accordingly, the principal strength of the present case lies in documenting a first presentation of giant infrahyoid TGDC in an elderly patient, with hoarseness and swallowing-related symptoms caused by secondary compression of the laryngeal cavity. From a clinical perspective, the present case suggests a relevant association between anatomical location, lesion size, and symptom pattern: the cyst was centered in the infrahyoid region, reached a giant size, and extended deeply enough to compress the adjacent laryngeal cavity, which plausibly explains the patient's hoarseness, foreign body sensation, and swallowing obstruction. However, a statistically meaningful correlation between cyst location, dimensions, symptoms, and patient age cannot be established from a single case report. In this setting, age is more likely to influence the diagnostic context than to determine the anatomical level of the cyst itself. In an elderly patient with a midline neck mass and hoarseness, the differential diagnosis should include not only TGDC but also mixed laryngocele, primary laryngeal malignancy, cystic metastatic cervical lymphadenopathy, branchial cleft cyst, cervical lymphatic malformation, dermoid or epidermoid cyst, ectopic thyroid tissue, and TGDC-associated carcinoma. This age-specific differential diagnosis differs from that in childhood, when congenital midline neck lesions are more readily considered. In elderly patients, however, congenital lesions such as TGDC may be overlooked, whereas adult laryngeal and other neck pathologies are often considered first. Therefore, maintaining diagnostic awareness of TGDC in older patients is clinically important, particularly when the lesion presents with hoarseness and imaging evidence of laryngeal compression. Laryngocele, also known as laryngeal saccule dilatation or laryngeal air hernia, is an abnormal cystic dilatation of the laryngeal saccule, communicating with the laryngeal lumen and filled with air (6). The cyst wall is lined with pseudostratified ciliated columnar epithelium (7). Laryngoceles are classified as internal, external, or mixed. Mixed laryngoceles involve both the larynx and neck, connected by an isthmus at the level of the thyrohyoid membrane, resembling a dumbbell shape (8). Given the similarity in symptoms, signs, and laryngoscopic findings, careful correlation of lesion location, laryngoscopic findings, and radiological appearance was crucial in differentiating TGDC from mixed laryngocele in this case. The lesion was located in the anterior midline neck around and below the hyoid bone, surrounded the hyoid bone on ultrasound, and appeared as a cystic low-density lesion on CT. CT demonstrated compression and narrowing of the adjacent laryngeal cavity rather than a primary air-filled laryngeal saccular lesion. These findings supported an infrahyoid TGDC producing secondary laryngeal compression rather than a primary laryngeal lesion. Postoperative histopathological examination confirmed the diagnosis of TGDC, and the favorable short-term outcome was supported by resolution of hoarseness and swallowing obstruction together with a normal laryngoscopic finding at the 3-month follow-up.

Preoperative evaluation of a cystic neck mass in an adult or elderly patient requires caution. Although TGDC is usually benign, carcinoma arising in a TGDC is rare but clinically important. FNAC may be useful in selected cases, particularly in adult or elderly patients, when the lesion shows mural nodules, calcification, solid enhancing components, suspicious cervical lymphadenopathy, concomitant thyroid lesions, or persistent diagnostic uncertainty. In the present case, preoperative FNAC was not documented in the available medical record. This is acknowledged as a limitation. However, cervical ultrasound showed no obviously abnormal cervical lymph nodes, CT showed no obvious primary pharyngeal or laryngeal mass within the scanned field, and postoperative histopathological examination supported TGDC with old hemorrhage and cholesterol crystal formation, without a reported histopathological diagnosis of carcinoma. Therefore, we emphasize that FNAC should be considered in future adult or elderly TGDC cases when suspicious features or diagnostic uncertainty are present.

Confirmation of normally located thyroid tissue is important before TGDC surgery because ectopic thyroid tissue may rarely represent the patient's only functioning thyroid tissue. In this case, CT showed thyroid-related structures in the normal cervical anatomical region, which reduced this concern. However, CT also incidentally identified a 14 mm × 12 mm nodular shadow with small calcifications in the lower region of the left thyroid lobe. Detailed thyroid function test results and dedicated thyroid nodule evaluation were not available in the scanned record. This issue has therefore been acknowledged as a limitation, and further thyroid evaluation and follow-up should be recommended clinically.

Regarding the surgical approach, open excision based on the Sistrunk principle was selected in this case. Although minimally invasive approaches, such as transoral robotic surgery and endoscopic hyoid-sparing TGDC excision (9), have been described, open surgery was considered more appropriate for this patient because the lesion was giant, closely attached to the hyoid bone, dumbbell-shaped, and partially extended posterior to the thyroid cartilage with significant laryngeal compression. Open surgery provided adequate exposure, allowed safe dissection around the hyoid bone and adjacent laryngeal framework, enabled complete removal of the cyst and involved hyoid segment, and helped reduce the risk of recurrence. During the procedure, the cyst was decompressed by aspiration to improve visualization and facilitate dissection. Postoperative follow-up confirmed resolution of hoarseness, phonation difficulty, foreign-body sensation, and swallowing obstruction, further supporting the diagnosis of TGDC causing secondary laryngeal compression.

The strengths of this report include a relatively complete clinical evidence chain, comprising physical examination, laryngoscopic evaluation, ultrasound, CT, intraoperative findings, histopathological confirmation, and short-term follow-up. In addition, the case highlights an uncommon first presentation of giant infrahyoid TGDC in an elderly patient with secondary laryngeal compression. However, several limitations should be acknowledged. First, this is a single-case report, and therefore no statistically meaningful correlation can be established between cyst location, lesion size, symptoms, and patient age. Second, preoperative FNAC was not documented, although FNAC may be useful in selected adult or elderly patients with TGDC, especially when suspicious imaging findings, concomitant thyroid nodules, or diagnostic uncertainty are present. Third, detailed thyroid function test results and dedicated thyroid nodule evaluation were not available in the scanned record. Although CT showed thyroid-related structures in the normal cervical anatomical region, future cases should include explicit thyroid function assessment and dedicated thyroid evaluation before surgery. Fourth, the follow-up period was relatively short, and no objective postoperative voice analysis was performed. Longer follow-up is needed to monitor recurrence and the incidental thyroid-region nodule.

## Data Availability

The original contributions presented in the study are included in the article/Supplementary Material, further inquiries can be directed to the corresponding author.
